# Exploring healthcare workers’ experiences of managing patients returning to HIV care in Johannesburg, South Africa

**DOI:** 10.1080/16549716.2021.2012019

**Published:** 2022-01-17

**Authors:** Melanie A. Bisnauth, Natasha Davies, Sibongile Monareng, Helen Struthers, James A. McIntyre, Kate Rees

**Affiliations:** a Department of Public Health, Anova Health Institute, Johannesburg, South Africa; b Department of Community Health, School of Public Health, University of the Witwatersrand, Johannesburg, South Africa; c Department of Health Sciences, School of Public Health, University of the Witwatersrand, Johannesburg, South Africa; d Division of Infectious Diseases & HIV Medicine, Department of Medicine, University of Cape Town, Cape Town, South Africa; e School of Public Health and Family Medicine, University of Cape Town, Cape Town, South Africa

**Keywords:** Re-engagement, HIV, retention, organizational behavioural change, differentiated care

## Abstract

**Background:**

Retention of patients in HIV care is a critical barrier to reaching the UNAIDS 90–90-90 goals in South Africa. In January 2019, Anova Health Institute launched a campaign to encourage patients who had interrupted antiretroviral therapy to return to care. The Welcome Back campaign included training of health care workers and implementation of Médecins Sans Frontiers Welcome Services principles.

**Objective:**

The aim of this study was to explore the experiences of healthcare workers managing patients reinitiating antiretroviral therapy following training, including barriers and facilitators to implementation.

**Methods:**

Data were collected from six clinics. This study consisted of three components: 1) surveys; 2) semi-structured interviews and 3) reflexive feedback sessions. Each component covered staff attitudes and facility management of patients reinitiating antiretroviral therapy. A descriptive analysis was conducted of survey responses. A thematic approach was used to analyze interviews.

**Results:**

Thirty-six healthcare workers completed the survey and interview. Following analysis, feedback sessions were conducted with 99 healthcare workers. Twenty-two (61%) participants were lay counsellors. The majority of healthcare workers reported managing patients returning to care appropriately. However, barriers persisted: 9 (25%) responded that patients were sent to the back of the queue and that service providers continued to insist on transfer letters. Twenty-five (69%) responded they had seen/heard other healthcare workers act poorly towards returning patients after training. Many poor behaviours from healthcare workers stemmed from frustration with the clinical flow and their overburdened work environment. Many participants (78%) believed that the Welcome Back approach helped improve client-provider relationships.

**Conclusions:**

The Welcome Back approach supported healthcare workers to improve service provision for patients reinitiating antiretroviral therapy. Further support is needed to help providers consistently deliver services in line with the Welcome Back approach. Institutional level changes are required to implement patient-centred and trust-based models of care.

## Background

The South African antiretroviral therapy (ART) programme is the largest in the world [1,2]. In 2019, UNAIDS reported there were 7 500,000 people living with human immunodeficiency virus (HIV) in South Africa, of which 92% (6 900,000) had been diagnosed with HIV, 70% (5 200,000) were on ART and 92% (4 800,000) were virally suppressed [2]. In Gauteng province, 87% were diagnosed with HIV, 61% were on ART and 88% were virally suppressed [3]. According to a 2018 review, in South Africa only 63% of patients who initiated ART remained in care after four years [2,4]. As the South African ART programme has rapidly expanded over recent years, there has been a strong focus on achieving high initiation rates. However, a crucial need to focus on long-term retention remains. If the second 90 (90% of those who know their HIV status should be on sustained ART) is to be achieved, greater effort is needed to understand and tackle barriers to retention and return to treatment following interruptions. This will allow the development of effective interventions to re-engage people who have interrupted treatment [1–4].

Evidence shows that retention of patients within the South Africa HIV programme has been compromised by organizational and behavioural challenges that impact negatively on the delivery of quality services [4–6]. These challenges impact negatively on the way patients experience services and on their subsequent retention or re-engagement. Elements of service delivery that need to be improved to, support the retention and re-engagement of patients, include the clinic flow and service efficiency, drug availability and tolerability, and health care providers’ attitudes and behaviours towards service users.

### Barriers and facilitators influencing retention in care

Retention in care is defined as remaining connected to and engaged with medical care after initial access [6,7]. This is critical for optimal clinical outcomes [6,7]. However, significant losses occur at each stage of the continuum due to contextual, social and structural factors. Studies have explored multi-level barriers and facilitators influencing entry into, engagement in, and retention along the continuum of care [7]. Key individual barriers include factors such as reluctance to engage in HIV services while healthy, alternative healing systems, income, and distance to health facilities [6–8]. Health system factors often operate at a facility level, and include: rigid clinic policies; stock-outs of medication; stigma and discrimination, and negative patient-provider relationships [5–8].

Service providers are the backbone of the ART programme and an important contributor to some patients disengaging from care. Trust is key to building good interpersonal relationships between patients and providers. However high staff turnover, congested clinics, lack of continuity of care and pressured targets, create an environment where fostering this trust becomes difficult. A lack of trust can negatively affect retention in care and an individual’s likelihood of re-engaging with care after an interruption [1]. Patients’ care experiences at clinics can be affected by all cadres of clinical and non-clinical staff, including administrative staff and security [5]. Retention can also be compromised by insufficient numbers of skilled and trained staff [7]. Lack of empathy, psychological burnout, bias or discrimination, and disrespectful treatment can also be key factors in disrupting care [1,5,8]. Furthermore, studies have suggested that some patients distrust healthcare providers and the larger healthcare system in South Africa due to poor quality service delivery, long queues, drug stock-outs, poor attitudes and communication from staff, and lack of leadership [5–9].

Coming back to a clinic after a break in treatment can be overwhelming, and patients might anticipate or actually experience negative reactions from clinic staff; this serves as a further reason to remain disengaged from care [1,9]. Treatment interruptions are often part of the treatment journey for patients with chronic conditions. The onus is on healthcare workers (HCWs) to understand the challenges that each individual may face and accept these in a non-judgemental manner. Often, the organizational culture remains punitive, generating fear amongst patients, which pushes them towards remaining out of care for longer and more damaging periods of time [1,8,9].

### Addressing the gap in the healthcare system

Healthcare workers’ (HCWs’) attitudes and behaviour are an amenable and cost-effective opportunity for change in healthcare systems, where constraints due to limited physical resources are not easy to change [9–11]. HCWs’ behaviour can shift and it is worth investing in ways to change behaviour. By targeting low-cost behavioural interventions to specifically address HCWs-related barriers, the country may be able to strengthen quality of care [9,10]. HCWs can be encouraged to behave well towards their patients and normalize their ART treatment journeys. Research has suggested that behavioural interventions can be cost effective when including educational outreach, health reminders, and feedback sessions, as part of the overall intervention [9]. Combinations of interventions are most likely to change behaviour [9–11].

### The welcome back campaign intervention

Anova Health Institute is a non-governmental organization and United States Agency for International Development (USAID) funded supporting partner to the Department of Health (DoH) in five districts of South Africa. In January 2019, Anova launched a Welcome Back (WB) campaign to encourage ART patients who had interrupted treatment to return to care. The campaign consisted of training HCWs and non-clinical facility staff, using training modules developed by Médecins Sans Frontiers (MSF), and mobilising patients to return to care through mass media messaging [11]. HCWs were trained on the WB approach components. Following an introductory section about the frequency and common causes of patients disengaging from and returning to care, HCWs were introduced to the ‘Welcome Back Handshake’ developed by MSF [12]. This handshake introduced HCWs to five key client-centred behaviours to perform when interacting with a returning client. They included: 1) ***Welcome***: welcoming the patient to the service and making them feel valued; 2) ***Normalize***: normalizing the struggle that many individuals have with remaining on treatment and reducing patient guilt for having previously disengaged; 3) ***Acknowledge***: acknowledging in a positive way that patients had taken the decision to return to care and build upon that achievement; 4) ***Support***: providing individualized support to the patient to remain adherent to their treatment in the future and; 5) ***Empower***: empowering patients to take ownership of their treatment and care journey going forward [11].

All staff, across all cadres, were invited to attend a once-off training session that was repeated on multiple days to enable as many staff as possible to attend. Training was provided by either an experienced HIV clinician and public health specialist or clinical trainers, who had previously been trained. Ongoing site supervision was undertaken after the training to monitor implementation. Of our study participants, 35 out of 36 had attended the training, indicating high coverage. All cadres were invited to the training including clinicians, enrolled nurses, counsellors and administrative staff. Data review meetings were held at participating facilities, which incorporated reflexive feedback sessions. Ongoing site supervision was undertaken by management teams after the training to monitor implementation.

As part of an evaluation of the campaign, we interviewed HCWs that provided services to patients returning to care, after training and implementation of the WB campaign in public sector clinics in South Africa. The aim of this study was to explore the experiences of healthcare workers managing patients reinitiating antiretroviral therapy following training, including barriers and facilitators to implementation.

## Methods

### Study site

The study was conducted in six clinics in one administrative region of Johannesburg, Region E. This area, including Alexandra township, makes up about 14% of Johannesburg’s population, with approximately 700,000 inhabitants [13]. There are nine clinics in the region, including one community health centre that provides a wider range of services. Six of the clinics, that serve over 28,000 ART patients, began implementation of the WB approach in January 2019.

### Study design

Data were collected eleven months after implementation of the WB approach (November 2019). This mixed methods study consisted of three components; 1) Surveys were completed by HCWs, covering their own and their facility’s management of patients reinitiating ART; 2) Semi-structured interviews were conducted with HCWs that had completed the survey. Interviews covered staff attitudes and facility management of patients reinitiating ART in more depth and; 3) Reflexive feedback sessions were conducted to gain further insight into the study findings.

Study participants were recruited from six clinics, aiming to achieve representation across all cadres of HCWs. All HCWs, employed by Anova 18 years or older, who were involved in reinitiating ART were eligible for inclusion.

All eligible HCWs were invited to participate on a voluntary basis, and the names and contact details of those who agreed were shared with the research team.

### Healthcare worker surveys

Survey data was captured anonymously into a REDCap (Research Electronic Data Capture) database [14]. The tool had both check box answers where the participant could choose multiple responses (e.g. *Can you describe what happens when a visitor comes to the clinic for treatment?*), and free text responses which included HCWs’ attitudes and practices towards patients after treatment interruption (e.g. *Are there any parts of the welcome back campaign that are more difficult to implement?*).

### Qualitative: semi-structured healthcare worker interviews

The qualitative component aimed to investigate HCWs’ perceptions in more depth, to understand their attitudes and behaviours towards patients that had disengaged from care [15]. The semi-structured interviews explored contextual factors that could not be fully explored in the surveys.

### Reflexive feedback sessions

All HCWs from Anova and DoH at the six facilities were invited to participate in a voluntary feedback session to communicate and discuss findings from both the survey and interviews. The reflexive sessions served as a way to collect data about what HCWs thought about the findings. Participants were informed that the discussion would be recorded and the research team would take notes to add to the study. Participants signed an attendance register.

### Ethical approval

Ethical approval for the study was granted by the Human Sciences Research Council, Research Ethics Committee (HREC) (approval HREC Number: REC 3/22/08/18). Written consent was obtained for interviews.

### Data collection & management

Participants who completed the survey were invited to a face-to-face in-depth interview. All participants consented and were interviewed.

An interview guide was used that covered participants’ experiences including: 1) understanding and awareness of the purpose, aim and importance of the WB approach; 2) attitudes and behaviour toward patients after treatment interruption, and; 3) clinical practices, and experiences with reinitiating patients.

All interviews were audio recorded and transcribed following participant consent. Memos and journaling were used to maintain a process log, capture any observations and maintain a record of analytic decisions throughout data collection and analysis. Transcripts, demographic information, observations of participant behaviour and journal memos were entered into NVivo 12.0 (QSR International Pty Ltd., 2018). In order to explore any preconceived notions from the research team, reflective journaling was used.

## Analysis

The descriptive surveys and semi-structured interviews were analyzed separately. A descriptive analysis was conducted of the survey responses. We reported frequencies and percentages for each response.

A thematic approach was used to analyze interviews. The process of analysis began with open coding directly from the data. Codes were grouped into categories. Three researchers in the team coded individually. Consensus between two researchers in the team was reached at the third version of the coding scheme. The categories were reduced to identify key themes and any commonalities and differences between cadres of HCWs were noted.

## Results

Thirty-six (84%) of 43 HCWs employed by Anova at the six facilities participated and completed the survey and interview. The duration of interviews ranged from 30 to 45 minutes. A total of 99 participants across the six clinics, from both Anova and DoH, attended the reflexive feedback sessions.

Twenty-two (61%) participants were lay counsellors. The lay counsellors were heavily represented because they perform a key function in the WB approach. Lay counsellors are often the first point of contact for returning patients, assisting them with navigation of the clinic system, linking them to other relevant clinical and non-clinical staff, and providing enhanced adherence counselling and follow-up support (Table 1).

**Table 1.** Occupation of participants in the welcome back campaign evaluation

Variable	Sub-category	Frequency
Occupation (36)	Lay counsellors	22
	Data Capturer	4
	Administrative Clerk	2
	Nurses	8

Thirty-five (97%) participants had had training on the WB approach and were familiar with WB components.

### Facility set-up of services

Twenty-eight (78%) participants responded that there is a specific person, such as a lay counsellor, designated to help patients navigate the facility and processes when returning to care. Twenty-seven (75%) participants reported changes to clinical flow, with facilities identifying one clinician to reinitiate all patients.

### Provider practices

The majority of HCWs responded that patients returning to care were managed appropriately. The most common responses included: 29 (81%) said they welcomed, 27 (75%) said they encouraged those that returned, 26 (72%) said they offered adherence support, and 19 (54%) said they empowered patients. However, only 6 (17%) of participants reported that they normalized the struggle with remaining on treatment and reduced patient guilt for having previously disengaged. Participants further demonstrated, following training, high levels of awareness by identifying unhelpful behaviours when asked, ‘*can you provide any examples of negative practices when people return to care?’* The following incorrect actions were correctly identified as negative practices: 26 (72%) HCWs selected sending a patient to the back of the queue, 26 (72%) selected criticize/judge/punish the returning client, and 23 (64%) selected insisting on a transfer letter.

Interviews revealed that most understood that patients face many difficulties accessing care, and empathized with their challenges. Responses also indicated that many supported returning patients by providing education about the risks of developing treatment resistance and the importance of viral load suppression.

However, some poor practices still persisted. Nine (25%) HCWs reported they still insisted on a transfer letter. Participant responses revealed returning patients still waited longer to be serviced by healthcare providers, *‘Staff members* [still] *shout at patients for refusing treatment and make them wait longer to be helped’* [IDI 4].

### Influences on provider practices

HCWs attending reflexive sessions thought there were many patients that move between facilities or ‘shop around’ when accessing ART which leads to distrust from HCWs. Many poor behaviours and attitudes stemmed from frustration with the clinical flow and feeling that the WB approach added to their overburdened work environment. *‘The WB messaging promises clients that when they come back they don’t have to wait; they can fast track through but our resources have not changed. We are still busy and hope that they understand we welcome them but they need to be patient with us too’* [IDI 12].

Participants were frustrated when patients without appointments disrupted their workflow, leading to increased waiting times for patients adhering to set appointments. This disruption led to patients becoming impatient and leaving the facility without receiving care, and HCWs subsequently feeling resentment toward returning patients. Participants said, they informed patients if they will miss an appointment to *‘arrange* [beforehand] *with their appointment card and have someone that they trust, pick up the medication before they leave’* [IDI 14] to avoid future treatment interruptions.

Although HCWs understood what the appropriate practices are, they struggled to adopt all the components of the WB approach due to the operational and organizational culture of the facility as a whole: 25%, nine participants, from three facilities, responded that patients were still being sent to the back of the queue and service providers continued to insist on transfer letters.

Twenty-five (69%) HCWs responded that they had seen or heard other HCWs act poorly towards returning patients, even after training was conducted, which demonstrated difficulty in shifting provider behaviour. Attitudes and behaviours discussed included lecturing and judging, and refusing patients ART if previous treatment could not be proven, *‘Yes, defaulting patients from ART treatment are refused treatment and* [are] *told to go back to their clinic where they started their treatment’* [IDI 16]. Many participants expressed that these poor attitudes and behaviours resulted from working within highly demanding and rigid work environments.

### Provider attitudes toward returning patients

Interviews with lay counsellors across all six facilities revealed that they aimed to ensure all patients were easily accessing treatment without blame. A lay counsellor expressed her belief that patients deserve to be treated with dignity, respect and integrity: *‘To ensure all HIV patients are easily accessing treatment without blame, giving a second chance, allowing them to come back to* [the] *facility without judgment’* [IDI 12].

Participants highlighted the importance of encouraging patients to voice their adherence challenges to enable the provision of support to overcome these barriers. A lay counsellor expressed the importance of understanding patients’ stories when managing their care, not making them feel guilty and explaining processes to them: *‘Allow them to tell us their challenges with taking treatment and how can we change that to overcome those challenges’* [IDI 9]. A professional nurse stated: *‘It is braveness on its own to have insight and come for their medication all over again. You may not know what caused them to stop’* [IDI 12].

Communication between lay counsellors and data capturers needed to be strengthened when it came to checking the medical history of patients, including what treatment they were on, where they initiated treatment, and how many times have they interrupted treatment. Participants suggested that streamlining communication lines for counsellors would increase the amount of time they spent with their patients. Some lay counsellors felt, *‘I am repeating the clients’ story multiple times instead of spending time counselling. There is a need to feel part of the team and I feel scared to report when a patient does not reinitiate and leaves’* [IDI 13] which demonstrated the counsellors fear to speak up about her challenges.

A lack of political will was noted in researchers’ notes from reflexive sessions that alluded to the HCWs’ need for better support and direction when implementing WB. Communication challenges between levels of staff seemed to interrupt the ability to strengthen the clinical flow.

Reflexive sessions revealed similarities between HCWs and support staff, both often felt blamed due to a lack of understanding from their patients. Participants expressed that patients often expected to be seen right away, even though they had missed their appointment date. An administrative clerk stated, *‘They don’t want to queue though we tell them with the counsellor they have to. They still expect to go straight to the room’* [IDI 28].

### Changes over time: Improvements with training

Some participants indicated a reduction in poor practices following implementation of WB. Both nurses and lay counsellors expressed how important it is that patients be empowered to take responsibility for their own health.

Twenty-eight participants (78%) believed that the WB approach helped to improve client-provider relationships. After implementation of the WB components, 27 (75%) HCWs’ believed patient experiences had improved and 26 participants (72%) believed staff treatment of patients had improved. Many HCWs felt that it should be continued as a practice within facilities: *‘The welcome back should be ongoing as this makes* [it] *easy for patients to communicate freely without having fear of being treated badly’* [IDI 9].

## Discussion

This study highlights key barriers that influence how HCWs support ART patients who have interrupted treatment, and explores how these are negotiated by service providers. The study findings could assist in developing better support systems for HCWs to foster positive interactions with patients who return to HIV care. The way that health systems and facilities are organized can influence provider behaviours, contributing to poor patterns of service delivery [8,15]. Even within complex health systems and resource constrained facilities, HCWs’ behaviour can be amenable to change, as seen amongst the participants of this study.

However, it is evident that poor attitudes and practices persisted after a comprehensive training intervention, despite HCWs displaying an understanding of their impact. For example, 25% of participants reported that re-engaging patients are sent to the back of the queue and the majority reported having seen patients treated badly by other HCWs. Insisting that relocating patients provide a clinic transfer letter before ‘allowing’ them to re-engage in care also remained a common damaging practice. Participants from three clinics continued to practice these counter-productive behaviours. A shift in organizational culture, driven by strong leadership, is needed to support staff to make sustainable changes to their behaviours in support of more client-centred care. Buy-in is needed from both frontline workers and management level staff if longstanding shifts in behaviour are to be achieved. There is a need for institutional level change to address the underlying organizational culture which continues to support punitive attitudes and behaviours towards patients who interrupt treatment [16]. There needs to be support for the principles of the WB campaign at all levels, and accountability, to maintain the shifts in provider behaviour over the longer-term. There remains a lack of political will to actively change the organizational culture to one of trust-based, patient-centred care. Functional clinic committees where patients can be involved in how facilities operate, and positive reinforcement for facilities where patient-provider relationships are good, should be encouraged.[17]

The majority of participants felt that people reinitiating ART were treated better since the onset of the campaign. Additional support can be offered to encourage re-engaging patients through task-shifting of re-engagement processes to other cadres of staff. Decreasing the number of patient interactions required for clinical staff may improve staff attitudes and decrease punitive responses.

Patients need to be made aware of their rights in terms of accessing healthcare services and re-engaging in care. Potential patients need to be empowered to insist on access to treatment where barriers are put in their way by unhelpful clinical or non-clinical staff. Education needs to be framed within positive language and not create conflict within client-provider partnerships, so that it does not perpetuate the culture of fear and blame that still predominates in many facilities. Messaging should stress the importance of working as a team. Whilst patients are empowered to know their rights, they should also be encouraged to be understanding of staff working in difficult and busy environments so that realistic expectations can be created.

Many poor attitudes and behaviours from HCWs stem from frustration with the clinical flow and additional services added to their overburdened work environment. It is important to find a balance between the information provided to patients and what the facilities can execute. A once-off training intervention is insufficient to shift entrenched HCWs’ attitude of resentment about their working environment. More work is needed to persuade HCWs that positive patient-provider relationships will result in a lower work burden because more patients will be stable, effectively retained in care, and thus become eligible for the decongesting options of multi-month dispensing and differentiated care models [18].

Research has suggested that differentiated services are highly acceptable and have good outcomes [15]. Differentiated care can serve as a tool for stable patients, decentralising care and reducing clinic attendance burden for healthy individuals adhering to ART [15]. When individuals disengage from care, they are no longer eligible for differentiated services because of being defined as not stable on treatment. Eligibility for access to differentiated care, which in turn supports retention and decongestion of facilities, should be used as a motivator to support patients to stay in care [15]. HCWs also need to acknowledge their role and power in supporting patients to access these decongesting options by providing quality and non-punitive care from the outset [16]. With fewer negative patient experiences, more patients would attain viral suppression, easing up the strained working environment. Further research is needed to explore the different rates of differentiated care enrolment in facilities with poor and good client-provider relationships – establishing a convincing evidence base to show that building trust and treating patients well actually reduces workload.

The WB approach should be integrated into all healthcare facilities including messaging around why patients disengage, to better identify barriers to retention and normalize treatment interruptions so that punitive attitudes are replaced by understanding and support. Without political will and managerial buy-in around shifting HCWs’ attitudes, there is little onus on providers to change their behaviour – the punitive approaches that are still prevalent need to be addressed by senior authorities in a positive manner. The system needs to be responsive to patients and ensure that frustrations are addressed in a time and behaviour sensitive manner, to reduce future disengagements.

In light of these findings, we recommend clinic managers and policy makers focus on ensuring a supportive environment for both HCWs and their re-engaging clients. This includes clear messaging to HCWs that punitive responses will not be tolerated, and HCWs will be held accountable for their treatment of clients. This should be accompanied by ongoing education about why clients disengage, how to support re-engagement, that more positive client-provider relationships lead to better retention, and subsequently lower burden in the long run. Processes should be set up that allow re-engaging clients to be managed efficiently, without disrupting workflows. In Johannesburg, we recommend that the WB approach be continued, and emphasis placed on integration into existing systems.

### Limitations

Potential reporting bias should be acknowledged as a risk in this study because participants knew what they should have been practicing and may have feared judgment if they reported negative behaviours. However, some questions were intended to try and get around this (e.g. *have you seen people acting negatively towards patients restarting ART?*). In addition, surveys and interviews were not conducted prior to the intervention to see if trends had changed.

A limitation of the study was that only Anova-funded staff were formally surveyed, although DoH staff did engage in the reflexive feedback sessions, adding breadth to the overall data. For the quantitative survey, a limitation was that 84% of Anova staff took the survey and this may have caused selection bias, as there is a possibility those who took on the WB philosophy were more likely to choose to complete the survey. A limitation of the qualitative work is that the team did not engage one cadre of informal gate keepers, security guards. Security staff are often the patients’ first initial contact when re-engaging with care at facilities in Johannesburg. Though administrative clerks were included, we recommend further exploration of the behaviours and practices of informal gate keepers including security, who have a large impact on patients’ initial experiences when re-engaging in care.

## Conclusion

In conclusion, the WB approach did support HCWs to improve their service provision to patients reinitiating ART, however, further support is needed for providers to help them consistently deliver good quality services. There needs to be institutional level change in order to implement truly patient-centred and trust-based models of care. Greater political will is needed to permanently engrain these shifts within South Africa’s health system.

## Paper context

Re-engaging clients who previously disengaged from HIV treatment programmes is critical. Certain health care workers’ attitudes and behaviours impact on the likelihood of retention and re-engagement. After training health care workers on positive re-engagement approaches, we found healthcare workers need ongoing support. Management should focus on creating an enabling environment for clients and healthcare workers, improving accountability and communication, fostering positive client-provider relationships and establishing clear client re-engagement processes that avoid disrupting existing workflows.
